# Dataset on permeability of wings from owls and non-silently flying birds

**DOI:** 10.1016/j.dib.2023.109825

**Published:** 2023-11-19

**Authors:** Thomas F. Geyer, Thomas Windisch, Christoph Fritzsche, Ennes Sarradj

**Affiliations:** a Technical Acoustics Group, Brandenburg University of Technology Cottbus - Senftenberg, Siemens-Halske-Ring 15A, 03046 Cottbus, Germany; b Leibniz-Institute for Solid State and Materials Research, Helmholtzstraße 20, 01069 Dresden, Germany; c Landesamt für Umwelt, Landwirtschaft und Geologie, Pillnitzer Platz 3, 01326 Dresden, Germany; d Institute of Fluid Mechanics and Engineering Acoustics, Technische Universität Berlin, Einsteinufer 25, 10587 Berlin, Germany

**Keywords:** Owl wing, Airflow resistivity, Permeability, Silent flight, Acoustics

## Abstract

The very soft and flow-permeable plumage is among the special adaptations of the owl that the silent flight is attributed to. Using a specially designed apparatus that provides a low-speed volume flow of air through a small sample of porous material, measurements of the air flow permeability were performed in accordance to ISO 9053 on a total of 39 prepared wing specimen from six different bird species, including three species of silently flying owls and three non-silently flying bird species. The resulting data set described in the present paper contains the static airflow resistance measured at different positions on the wing.

Specifications Table

**Table:** 

Subject	Acoustics and Ultrasonics
Specific subject area	Characterization of acoustic material properties of bird wings.
Data format	Raw, Analyzed
Type of data	Table, Image, Graph
Data collection	Measurements of the static airflow resistance were performed according to ISO 9053 at up to four locations on 39 prepared wing specimen belonging to six different bird species. This included three owl species known for their quiet flight and three other bird species. The airflow resistance is calculated as the ratio of the static pressure difference across the prepared wing, which was measured using differential pressure transducers, to the volume flow through the sample, which was provided by a small radial fan. The measurement device contained a special measurement head that allowed to perform in-situ measurements. The data were recorded using a National Instruments data acquisition module, and calculations were performed using in-house software.
Data source location	Technical Acoustics Group, Brandenburg University of Technology Cottbus - Senftenberg, Siemens-Halske-Ring 15A, 03046 Cottbus, Germany
Data accessibility	Repository name: Mendeley Data Data identification number: 10.17632/ndcndtstcf.1 Direct URL to data: https://data.mendeley.com/datasets/ndcndtstcf/1

## 1 Value of the Data


 

• The data presents the static airflow resistance measured on a set of prepared owl wings and that measured on wings of other, non-silently flying birds.
• The data provides evidence that the airflow resistance of owl feathers is different compared to that of other bird feathers.
• The data can be used to motivate or validate research on technical wings and airfoils modified with flow-permeable material for flow noise reduction.

## 2 Data Description

The dataset [1] consists of a comma-separated values (CSV) file named “dataset_wingper- meability.csv” that contains 149 rows and six columns. The first row contains the header and the remaining 148 rows contain the raw data. The first column specifies the species (e.g. Barn owl, Tawny owl, Long-eared owl, Common buzzard, Eurasian sparrowhawk or Common pigeon) as a string format. The second column specifies the corresponding binomial name (e.g. Tyto alba, Strix aluco, Asio otus, Buteo buteo, Accipiter nisus or Columba livia) as a string format. The third column contains the number of the wing, which have simply been numbered consecutively from 1 to 39 as integer data type. The fourth column specifies whether it is a left wing or a right wing, again as a string value. The next column contains the measurement position on the wing, which have been numbered from 1 to 4 as integers. The final column then contains the measured airflow resistance *R* in Pa s/m^3^ as floating point values. The csv-file uses Unicode (UTF-8) character encoding and the fields are separated by commas.

The dataset also contains an image as a Portable Document Format (PDF) file named “measurement_positions.pdf”. It shows a photograph of a prepared owl wing indicating the four measurement positions.

The third file of the dataset is a graph in the Portable Document Format (PDF) file named “wingpermeability_boxplot.pdf”. It shows the measured airflow resistances contained in the dataset as a box plot.

## 3 Experimental Design, Materials and Methods

The silent flight is attributed to three different adaptations of the plumage of owls, which have first been described in that regard by Graham in 1934 [1]: (1) a comb-like structure at the leading edge of the wings, (2) long and soft fringes at the trailing edge of the wings and (3) a soft and flow-permeable down that covers the wings. These adaptations have served as motivation for many researchers that try to transfer the underlying physical mechanisms responsible for the noise reduction to technical airfoils and wings. The physical properties of the plumage of owls have been studied and published in various biological journals [2], [3], [4], [5], [6]. However, although the permeability of owl wings has been described qualitatively in many of these studies, it has never been measured. The dataset described in the present paper contains measured airflow resistance values of a large set of prepared bird wings.

In total, 39 prepared wings of different bird species were used in the study. This included wings from the Barn owl (*Tyto alba*), the Tawny owl (*Strix aluco*) and the Long-eared owl (*Asio otus*), representing the silently flying species, as well as wings from the Common buzzard (*Buteo buteo*), the Eurasian sparrowhawk and the Common pigeon (*Columba livia*) as representatives of birds that do not fly silently. The wing specimen were not specially prepared for these measurements, but were provided by the *Senckenberg Naturhistorische Sammlungen Dresden* and the *Institute for Biology 2 at the RWTH Aachen University*. It is assumed that the preparation has no effect on the airflow resistance. An overview of the wing specimen is given in Table 1.

**Table 1:** Specimen used for air flow resistance measurements.

Bird	Binomial name	Wings examined	Total measurement positions
Barn owl	*Tyto alba*	15	55
Tawny owl	*Strix aluco*	7	28
Long-eared owl	*Asio otus*	2	5
Common buzzard	*Buteo buteo*	8	32
Eurasian sparrowhawk	*Accipiter nisus*	2	8
Common pigeon	*Columba livia*	5	20

The permeability of an open-porous medium can be described using the so-called static airflow resistance *R* of the material, which can be calculated as

$$R=\frac{\Delta p}{q_{v}}=\frac{p_{+}-p_{0}}{q_{v}},$$

where *Δp* is the pressure difference across the porous sample, *p_+_* is the positive air pressure, *p_0_* is the ambient air pressure and *q_v_* is the volume flow through the sample. The airflow resistance is commonly measured according to ISO 9053 [7] on cylindrical samples of the porous material with a minimum diameter of 0.095 m and a thickness *Δx*. As described in [7], measurements of the airflow resistance have been performed at different flow speeds. Using the results from these measurements, a linear interpolation of the airflow resistance as a function of speed was computed and the extrapolated value at zero flow speed was taken as the final measurement result.

**Fig. 1:**
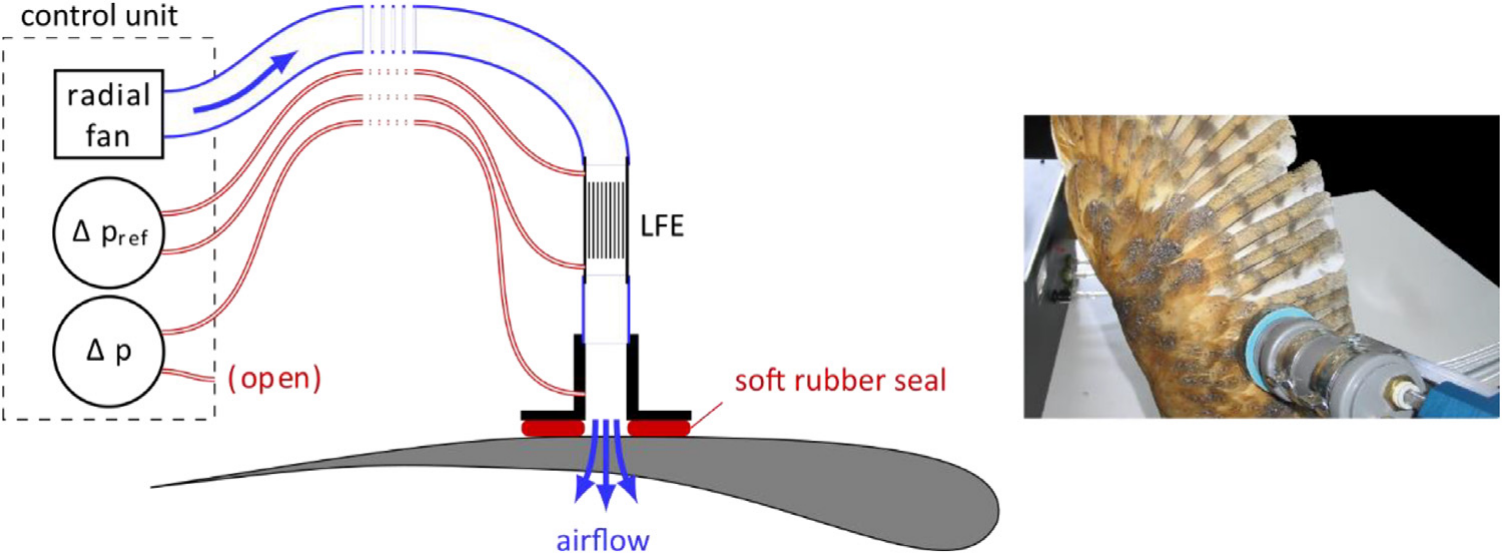
Apparatus for measuring the air permeability of wings. The fan produces an airflow through the laminar flow element (LFE), the measuring head and the test specimen. Differential pressures Δp_ref_ and Δp are measured using pressure transducers. The control unit sets the airflow velocity and computes the resulting airflow resistance R. The photograph shows a measurement on a specimen of Tyto alba.

For the intended measurements on wing specimen, a special apparatus was constructed that provides a constant low-speed airflow over a small sample area. This apparatus uses a measuring method that is based on the comparison of the unknown airflow resistance with a known airflow resistance, as shown in Fig. 1. To this end, a variable airflow is generated by a small radial fan. This airflow is lead through a so-called Laminar Flow Element (LFE) with a known resistance *R*_ref_. The LFE used here is a Fleisch pneumotachograph with a maximum flow rate of 2.4 l/min, which consists of a number of parallel capillary tubes. The pressure difference *Δp*_ref_ across the LFE is measured by a differential pressure transducer (type Setra D260), which converts it into an electric signal and has a maximum measuring range of 100 Pa and an accuracy of 1.0 % full scale. After passing the LFE, the airflow is fed into the sample by means of the measuring head and released into the surrounding atmosphere. The pressure difference *Δp* across the sample is recorded by another pressure transducer of the same type, but with a variable measuring range between 25 Pa and 250 Pa. The output voltages from both transducers are measured using a National Instruments USB-6008 data acquisition board that also provides the control voltage for the fan. The resulting airflow resistance is then calculated from the measured pressure differences according to

$$R=R_{\text{ref}}\frac{\Delta p}{\Delta p_{\text{ref}}}.$$

The calculation was performed with in-house software, which is also used to keep the flow velocity constant.

Since it was intended to measure the airflow resistance of the bird wings without damaging them, some changes needed to be made to the measurement procedure described in [7]. Instead of using a cylindrical sample holder, which would require to cut the wings into cylindrical samples to fit in the holder, an adapter was designed and constructed that allowed the performance of in situ measurements. The adapter consisted of a measurement head with an inner diameter of 20 mm equipped with a soft, flow-impermeable sealing. For the measurement, the measurement head is placed upon the prepared bird wing and the sealing prevents air leakage between the measurement head and the wing surface. Fig. 1 shows a schematic of the measuring head placed upon a bird wing.

**Fig. 2:**
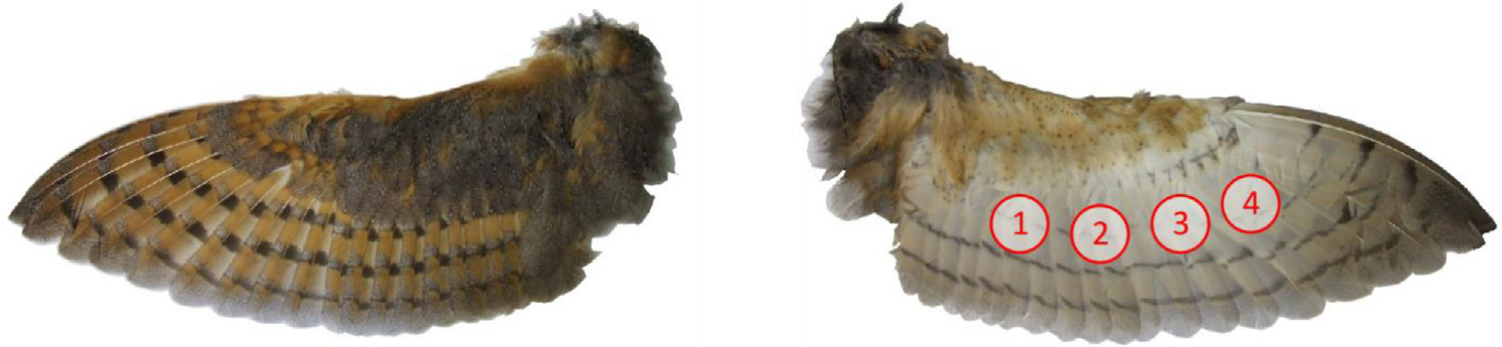
Photograph of the distal (left figure) and the proximal (right figure) side of one of the Barn owl wings used in the study (the markers show the approximate locations of the airflow resistance measurements).

**Fig. 3:**
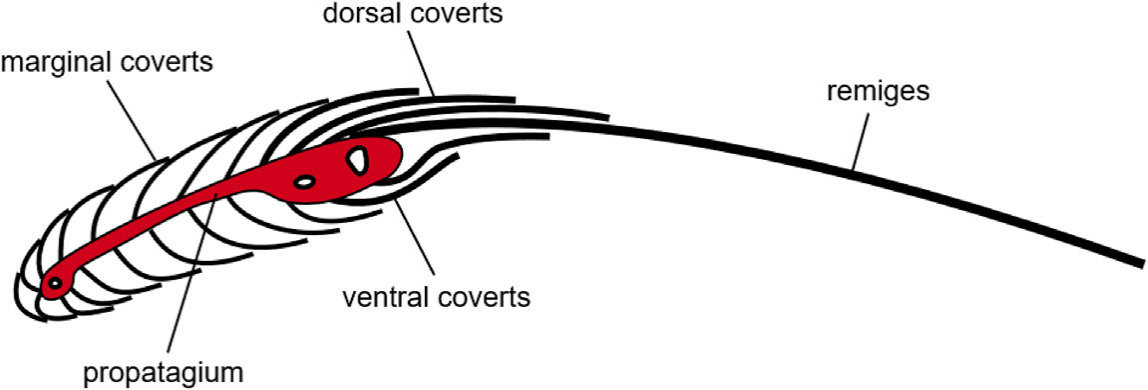
Schematic of a section through a bird wing at the level of the lower arm (adapted from [8]).

For each wing specimen, airflow resistance measurements were conducted at up to four positions over the wing, which are shown exemplarily in Fig. 2. As a part of the wing close to its leading edge contains bones, muscles and connective tissue (the so-called propatagium), which are impervious to air, only measurement locations located on the aft part of the wing, on its bottom surface, were selected. Fig. 3 shows a schematic of a section through a bird's wing. It was assumed that the airflow resistance does not vary between different remiges, and hence the positions were not assigned to specific primaries and secondaries. However, at every measurement position at least two layers of feathers were present. Although it is difficult to exactly quantify the resulting error of this method, it should be noted that the test procedure detailed in [7] is proven and tested. Especially the extrapolation of the measured airflow resistance values provides an adequate statistical significance.

**Fig. 4:**
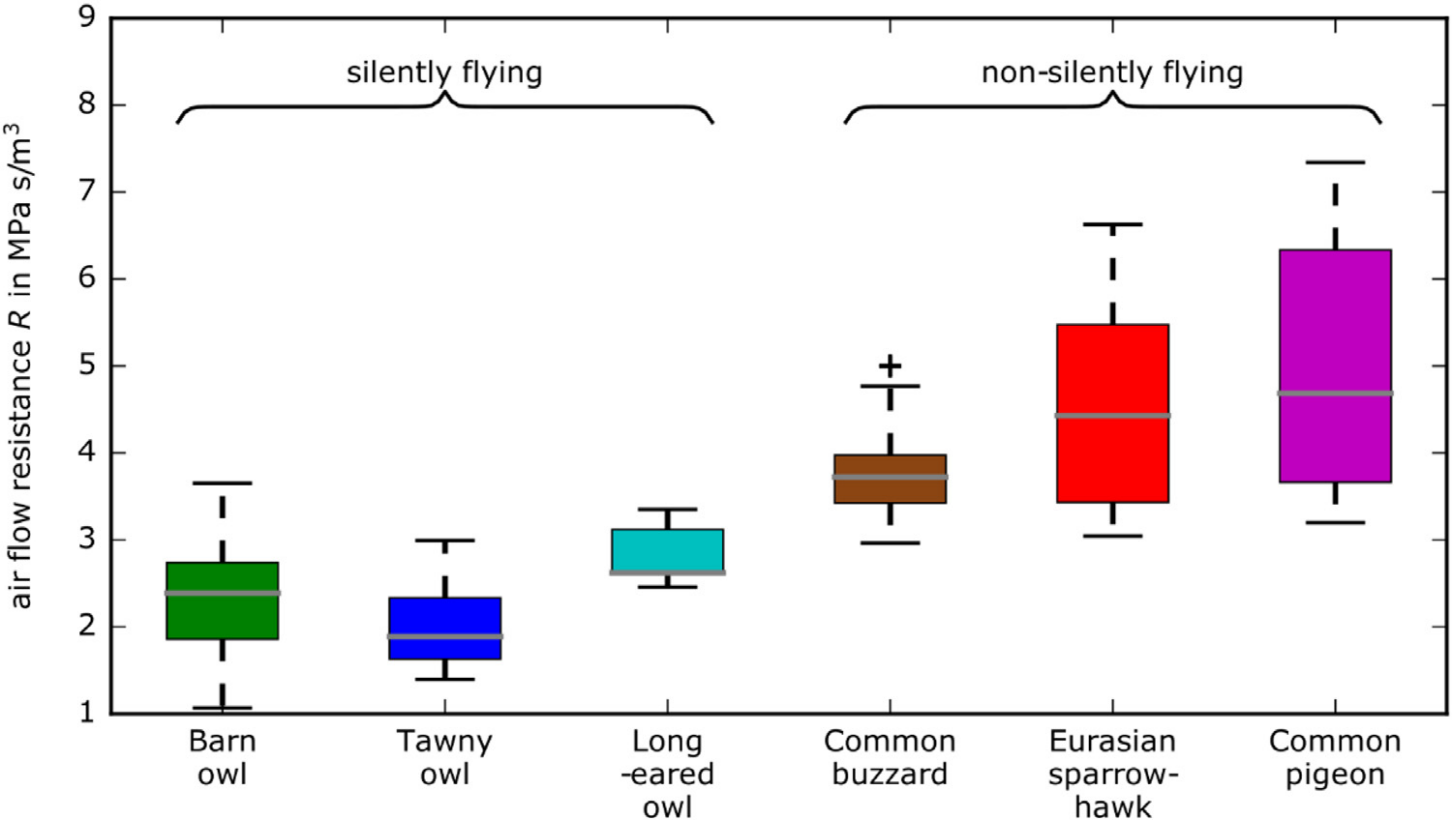
Resulting airflow resistances for the different bird wings.

The resulting data are available from the online repository [1] as well as in the Appendix. Additionally, the results are presented in Fig. 4 as a box plot, showing the median values, the minima, maxima and the first and third quartiles of the measured airflow resistances.

## Limitations

Not applicable

## Ethics Statement

The authors have read and follow the *ethical requirements* for publication in Data in Brief and confirm that the current work does not involve human subjects, animal experiments, or any data collected from social media platforms.

## CRediT Author Statement

**Thomas Geyer:** Formal analysis, Writing- Original draft preparation. **Thomas Windisch:** Investigation, Methodology. **Christoph Fritzsche:** Methodology, Data curation. **Ennes Sarradj:** Conceptualization, Formal analysis, Methodology, Software, Supervision, Resources, Funding acquisition, Writing- Original draft preparation.

## Figures and Tables

**Table 2:** Measured air flow resistance of the bird wings.

Species	Binomial name	Wing number	Side	Measurement position	Value (Pa s m^−3^)
Barn Owl	Tyto Alba	1	left	1	2542780,989
Barn Owl	Tyto Alba	1	left	3	1970893,606
Barn Owl	Tyto Alba	2	right	1	2015978,84
Barn Owl	Tyto Alba	2	right	2	3650510,976
Barn Owl	Tyto Alba	2	right	3	2637738,702
Barn Owl	Tyto Alba	2	right	4	1533212,585
Barn Owl	Tyto Alba	3	left	3	1258729,376
Barn Owl	Tyto Alba	3	left	4	1941889,223
Barn Owl	Tyto Alba	4	right	1	1977736,071
Barn Owl	Tyto Alba	4	right	3	1749187,603
Barn Owl	Tyto Alba	4	right	4	1413352,804
Barn Owl	Tyto Alba	5	right	1	2007756,851
Barn Owl	Tyto Alba	5	right	2	2549314,575
Barn Owl	Tyto Alba	5	right	3	3312561,784
Barn Owl	Tyto Alba	5	right	4	3037478,211
Barn Owl	Tyto Alba	6	right	1	2811699,507
Barn Owl	Tyto Alba	6	right	2	3647161,327
Barn Owl	Tyto Alba	6	right	3	2684771,145
Barn Owl	Tyto Alba	6	right	4	1819454,65
Barn Owl	Tyto Alba	7	left	1	2652948,705
Barn Owl	Tyto Alba	7	left	2	3101974,125
Barn Owl	Tyto Alba	7	left	3	2613345,376
Barn Owl	Tyto Alba	7	left	4	2782957,705
Barn Owl	Tyto Alba	8	left	1	1784507,807
Barn Owl	Tyto Alba	8	left	2	2565575,829
Barn Owl	Tyto Alba	8	left	3	2692522,962
Barn Owl	Tyto Alba	8	left	4	2165147,375
Barn Owl	Tyto Alba	9	right	1	1741611,225
Barn Owl	Tyto Alba	9	right	2	3244480,2
Barn Owl	Tyto Alba	9	right	3	1992297,453
Barn Owl	Tyto Alba	9	right	4	1455678,471
Barn Owl	Tyto Alba	10	right	1	1824260,859
Barn Owl	Tyto Alba	10	right	2	3356419,81
Barn Owl	Tyto Alba	10	right	3	1774277,741
Barn Owl	Tyto Alba	10	right	4	2475869,004
Barn Owl	Tyto Alba	11	left	1	1893360,157
Barn Owl	Tyto Alba	11	left	2	2602599,484
Barn Owl	Tyto Alba	11	left	3	2067622,455
Barn Owl	Tyto Alba	11	left	4	2676060,171
Barn Owl	Tyto Alba	12	left	1	3097292,741
Barn Owl	Tyto Alba	12	left	2	2695869,608
Barn Owl	Tyto Alba	12	left	3	2020893,686
Barn Owl	Tyto Alba	12	left	4	1937604,824
Barn Owl	Tyto Alba	13	right	1	3171077,889
Barn Owl	Tyto Alba	13	right	2	2271659,177
Barn Owl	Tyto Alba	13	right	3	2833561,397
Barn Owl	Tyto Alba	13	right	4	3009307,846
Barn Owl	Tyto Alba	14	left	1	2422391,713
Barn Owl	Tyto Alba	14	left	2	3009996,589
Barn Owl	Tyto Alba	14	left	3	2057967,539
Barn Owl	Tyto Alba	14	left	4	2388893,005
Barn Owl	Tyto Alba	15	right	1	1066665,746
Barn Owl	Tyto Alba	15	right	2	1725633,804
Barn Owl	Tyto Alba	15	right	3	1592095,454
Barn Owl	Tyto Alba	15	right	4	1727922,56
Tawny Owl	Strix Aluco	16	left	1	2595054,548
Tawny Owl	Strix Aluco	16	left	2	2983598,177
Tawny Owl	Strix Aluco	16	left	3	2360259,6
Tawny Owl	Strix Aluco	16	left	4	2459883,693
Tawny Owl	Strix Aluco	17	left	1	1742545,349
Tawny Owl	Strix Aluco	17	left	2	1530622,392
Tawny Owl	Strix Aluco	17	left	3	1521014,788
Tawny Owl	Strix Aluco	17	left	4	2000227,814
Tawny Owl	Strix Aluco	18	right	1	2084162,588
Tawny Owl	Strix Aluco	18	right	2	2107694,841
Tawny Owl	Strix Aluco	18	right	3	1992561,39
Tawny Owl	Strix Aluco	18	right	4	1720139,24
Tawny Owl	Strix Aluco	19	left	1	1663631,067
Tawny Owl	Strix Aluco	19	left	2	1729486,485
Tawny Owl	Strix Aluco	19	left	3	1871551,808
Tawny Owl	Strix Aluco	19	left	4	1454243,764
Tawny Owl	Strix Aluco	20	right	1	1393865,418
Tawny Owl	Strix Aluco	20	right	2	1509019,376
Tawny Owl	Strix Aluco	20	right	3	1529332,559
Tawny Owl	Strix Aluco	20	right	4	1418070,567
Tawny Owl	Strix Aluco	21	left	1	2325673,323
Tawny Owl	Strix Aluco	21	left	2	2433413,78
Tawny Owl	Strix Aluco	21	left	3	1893615,122
Tawny Owl	Strix Aluco	21	left	4	2407817,817
Tawny Owl	Strix Aluco	22	right	1	2995174,649
Tawny Owl	Strix Aluco	22	right	2	1884601,767
Tawny Owl	Strix Aluco	22	right	3	2323658,595
Tawny Owl	Strix Aluco	22	right	4	1739683,291
Long-Eared Owl	Asio Otus	23	left	3	2458031,17
Long-Eared Owl	Asio Otus	23	left	4	3349791,692
Long-Eared Owl	Asio Otus	24	right	1	3121195,96
Long-Eared Owl	Asio Otus	24	right	3	2607537,848
Long-Eared Owl	Asio Otus	24	right	4	2622324,529
Common Buzzard	Buteo Buteo	25	left	1	3910110,849
Common Buzzard	Buteo Buteo	25	left	2	3916440,002
Common Buzzard	Buteo Buteo	25	left	3	3863856,287
Common Buzzard	Buteo Buteo	25	left	4	4117245,16
Common Buzzard	Buteo Buteo	26	right	1	3499283,838
Common Buzzard	Buteo Buteo	26	right	2	3743405,053
Common Buzzard	Buteo Buteo	26	right	3	4093079,423
Common Buzzard	Buteo Buteo	26	right	4	4389341,311
Common Buzzard	Buteo Buteo	27	right	1	4994451,426
Common Buzzard	Buteo Buteo	27	right	2	3772721,381
Common Buzzard	Buteo Buteo	27	right	3	3948117,382
Common Buzzard	Buteo Buteo	27	right	4	4767070,27
Common Buzzard	Buteo Buteo	28	right	1	3367260,677
Common Buzzard	Buteo Buteo	28	right	2	3303761,774
Common Buzzard	Buteo Buteo	28	right	3	3235381,029
Common Buzzard	Buteo Buteo	28	right	4	3164347,227
Common Buzzard	Buteo Buteo	29	right	1	4346957,76
Common Buzzard	Buteo Buteo	29	right	2	3704775,482
Common Buzzard	Buteo Buteo	29	right	3	2962616,025
Common Buzzard	Buteo Buteo	29	right	4	3872486,318
Common Buzzard	Buteo Buteo	30	right	1	3140802,249
Common Buzzard	Buteo Buteo	30	right	2	3674596,981
Common Buzzard	Buteo Buteo	30	right	3	3515720,891
Common Buzzard	Buteo Buteo	30	right	4	3815190,796
Common Buzzard	Buteo Buteo	31	right	1	3373554,942
Common Buzzard	Buteo Buteo	31	right	2	3533354,389
Common Buzzard	Buteo Buteo	31	right	3	3611849,274
Common Buzzard	Buteo Buteo	31	right	4	3231913,432
Common Buzzard	Buteo Buteo	32	right	1	3502482,182
Common Buzzard	Buteo Buteo	32	right	2	3439911,175
Common Buzzard	Buteo Buteo	32	right	3	4056779,597
Common Buzzard	Buteo Buteo	32	right	4	5008404,034
Eurasian Sparrowhawk	Accipiter Nisus	33	left	1	5781387,129
Eurasian Sparrowhawk	Accipiter Nisus	33	left	2	3539088,447
Eurasian Sparrowhawk	Accipiter Nisus	33	left	3	3044624,347
Eurasian Sparrowhawk	Accipiter Nisus	33	left	4	5375413,331
Eurasian Sparrowhawk	Accipiter Nisus	34	right	1	4955913,756
Eurasian Sparrowhawk	Accipiter Nisus	34	right	2	3109786,225
Eurasian Sparrowhawk	Accipiter Nisus	34	right	3	3901678,269
Eurasian Sparrowhawk	Accipiter Nisus	34	right	4	6628080,78
Common Pigeon	Columba Livia	35	left	1	3391658,478
Common Pigeon	Columba Livia	35	left	2	3683287,484
Common Pigeon	Columba Livia	35	left	3	6562336,123
Common Pigeon	Columba Livia	35	left	4	6744048,749
Common Pigeon	Columba Livia	36	left	1	4703108,549
Common Pigeon	Columba Livia	36	left	2	3600095,839
Common Pigeon	Columba Livia	36	left	3	6033003,161
Common Pigeon	Columba Livia	36	left	4	6123663,939
Common Pigeon	Columba Livia	37	left	1	3700876,311
Common Pigeon	Columba Livia	37	left	2	4535098,949
Common Pigeon	Columba Livia	37	left	3	3490426,912
Common Pigeon	Columba Livia	37	left	4	4800507,682
Common Pigeon	Columba Livia	38	right	1	3196805,472
Common Pigeon	Columba Livia	38	right	2	3987242,303
Common Pigeon	Columba Livia	38	right	3	7340913,264
Common Pigeon	Columba Livia	38	right	4	6323026,496
Common Pigeon	Columba Livia	39	right	1	4669025,327
Common Pigeon	Columba Livia	39	right	2	3361901,886
Common Pigeon	Columba Livia	39	right	3	7026088,93
Common Pigeon	Columba Livia	39	right	4	6370091,03

## Data Availability

Dataset on airflow resistance of wings from owls and non-silently flying birds (Original data) (Mendeley Data)

## Appendix

In addition to the online repository [1], the following Table 2 contains the measured data as described in the Data Description section.

## References

[1] Geyer T.F., Windisch T., Fritzsche C., Sarradj E. (2023). Dataset on airflow resistance of wings from owls and non-silently flying birds. Mendeley Data.

[2] Graham R.R. (1934). The silent flight of owls. J. R. Aeronaut. Soc..

[3] Mascha E. (1904). Über die Schwungfedern. Z. Wiss. Zool..

[4] Sick H. (1937). Morphologisch-funktionelle Untersuchungen über die Feinstruktur der Vogelfeder. J. Ornithol..

[5] Bachmann T., Klän S., Baumgartner W., Klaas M., Schröder W., Wagner H. (2007). Morphometric characterisation of wing feathers of the barn owl tyto alba pratincola and the pigeon columba livia. Front. Zool..

[6] Bachmann T., Wagner H., Tropea C. (2012). Inner vane fringes of barn owl feathers reconsidered: morphometric data and functional aspects. J. Anat..

[7] ISO 9053: Acoustics - materials for acoustical applications - determination of airflow resistance. Standard, International Organization for Standardization, 1993.

[8] Müller W., Patone G. (1998). Air transmissivity of feathers. J. Exp. Biol..

